# Light Baths

**Published:** 1901-01-12

**Authors:** 


### LIGHT BATHS.

A QUESTION which is being discussed at the present time and is certainly not without interest, is as to how far what we may call the middle rays of light have any therapeutic influence. Of course we all know about Finsen's light treatment, in which the light is derived either from the sun or the arc lamp, the aim being to obtain as large a quantity as possible of the actinic rays and to cut off in every way the rays coming from the red end of the spectrum; and on the other hand we all know the effects of heat applied in its various forms—hot water, hot air, or mere heat radiation—without any light at all. But there are many forms of apparatus in use in which the patient is exposed to a mixed radiation of heat, and light, and actinism (if we may be allowed for the moment to speak of these forms of radiant energy as "things"), and for which special claims are now being put forward, and the question is how far the claims of these "light baths" worked by incandescent electric lights will hold good. Neither the advocates of heat nor those who put their faith in actinism will admit that there is any special excellence in them. They are said to be sweat-baths and that is all. Those who use them, however, think otherwise. We still seem to be in darkness about the action of light so far as its middle rays are concerned.

